# In Vivo Anti-Inflammatory, Analgesic, Muscle Relaxant, and Sedative Activities of Extracts from *Syzygium cumini* (L.) Skeels in Mice

**DOI:** 10.1155/2022/6307529

**Published:** 2022-04-11

**Authors:** Abdur Rauf, Yahya S. Al-Awthan, Imtaiz Ali Khan, Naveed Muhammad, Syed Uzair Ali Shah, Omar Bahattab, Mohammed A. Al-Duais, Rohit Sharma, Md. Mominur Rahman

**Affiliations:** ^1^Department of Chemistry, University of Swabi, Swabi, Anbar 23430, Khyber Pakhtunkhwa (KP), Pakistan; ^2^Department of Biology, Faculty of Science, University of Tabuk, Tabuk, Saudi Arabia; ^3^Department of Biology, Faculty of Science, Ibb University, Ibb, Yemen; ^4^Department of Entomology, University of Agriculture, Peshawar, Khyber Pakhtunkhwa (KP), Pakistan; ^5^Department of Pharmacy, Abdul Wali Khan University, Mardan, Khyber Pakhtunkhwa (KP), Pakistan; ^6^Department of Pharmacy, University of Swabi, Swabi, Anbar 23430, Khyber Pakhtunkhwa (KP), Pakistan; ^7^Department of Biochemistry, Faculty of Science, University of Tabuk, Tabuk, Saudi Arabia; ^8^Biochemistry Unit, Chemistry Department, Faculty of Science, Ibb University, Ibb, Yemen; ^9^Department of Rasa Shastra & Bhaishajya Kalpana, Faculty of Ayurveda, Institute of Medical Sciences, Banaras Hindu University, Varanasi-221005, Uttar Pradesh, India; ^10^Department of Pharmacy, Faculty of Allied Health Sciences, Daffodil International University, Dhaka 1207, Bangladesh

## Abstract

In the current study, the folklore medicine, *Syzygium cumini*, was experimentally evaluated for anti-inflammatory, analgesic, sedative, and muscle relaxant effects. The extract and fractions of *S. cumini* were found safe up to 1000 mg/kg with no mortality, except for slight sedation as a minor side effect. Both, the extract and various fractions of *S. cumini* demonstrated significant inhibition (86.34%) of carrageenan-induced inflammation in mice. Acetic acid induced writhes were attenuated (*p* < 0.001) by *S. cumini* in a dose-dependent manner, except for the *n*-hexane fraction. The maximum effect was observed at a dose of 500 mg/kg in mice. The maximum muscle relaxant effect of all tested samples was recorded at a dose of 500 mg/kg bodyweight, where the percent inhibition exhibited by dichloromethane fraction was 82.34%, followed by chloroform fractions (71.43%) and methanolic extract (70.91%). Our findings validate the folklore medicinal claims of *S. cumini*, as an analgesic and anti-inflammatory agent.

## 1. Introduction 

**Figure 1:**
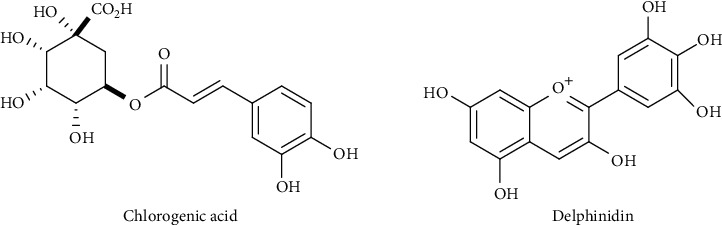
Chemical structures of major compounds isolated from *S. cumini*.

*Syzygium cumini* L. (Myrtaceae), commonly known as jamun, jambolan, black plum, and jambolao, is widely distributed in India, Africa, South America, and Pakistan [1–3]. This genus is famous for its multimedicinal usage as an antidiabetic and laxative [3]. Various parts of *S. cumini* have been reported for the presence of diverse bioactive natural compounds. *S. cumini* leaves are used for the treatment of constipation, dermopathies, leucorrhea, diabetes, and gastropathies, and its bark is used as an anthelmintic, carminative, and astringent. Apart from its role in diabetes, *S. cumini* is also used as diuretic and astringent [4–6]. *S. cumini* has been documented for diverse pharmacological properties, such as anti-inflammatory, antihyperglycemic, antioxidant, antimicrobial, and cardioprotective [7–11]. The crude extract of bark and its various fractions have been documented for excellent *α*-glucosidase, urease, and phosphodiesterase inhibitory potential [12]. Previous phytochemical investigations on *S. cumini* indicated the presence of various classes of bioactive compounds such as alkaloids, flavonoids, terpene, phenols, carbohydrates, saponins, and glycosides [12]. *S. cumini* leaves comprise of high level of flavonoids mainly myricetin, kaempferol, and quercetin. Other phytochemicals isolated from *S. cumini* include ferulic acid, ellagic acid, gallic acid, and chlorogenic acid [13–15]. Seed of *S. cumini* has been reported for the presence of *α*-terpineol, betulinic acid, eugenol, and other phenolic acids [16, 17]. The fruits are a rich source of anthocyanins such as delphinidin, cyaniding, and petudinine [17]. The stem of *S. cumini* is mainly composed phenolic acids, flavonoids, and terpenes [6, 16]. The major chemical constitutes isolated from *S. cumini*, chlorogenic acid and delphinidin (Figure 1), might be involved in its various biological actions. Considering the multimedicinal usage of *S. cumini*, the current study was designed to evaluate its crude extracts and various fractions for in vivo anti-inflammatory, analgesic, muscle relaxation, and toxicological effects.

## 2. Materials and Methods

### 2.1. Plant Materials

The plant materials were collected in the month of July, 2020, from the local area of Anbar, Swabi, Khyber Pakhtunkhwa, Pakistan (GPS coordinates latitude 34°53′40.41″N, longitude 72° 1′42.48″E). The plant specimen was identified and authenticated by Dr. Muhammad Ilyas, Department of Botany, University of Swabi, Swabi, Khyber Pakhtunkhwa, Pakistan. The specimen was tagged under voucher number UOS/Bot-103. The specimen was stored in the same department.

### 2.2. Preparation of Extract

*S. cumini* stem (10.76 kg) was washed with tap water to remove dust particles. The washed plant materials were dried under shade for 20 days. The dried plant material was ground with a grinding machine. The plant material was soaked in commercial grade methanol for 18 days until the extraction of polar and nonpolar compounds was almost complete. The methanolic extract was concentrated under low pressure and temperature to prevent the decomposition of heat-labile molecules. The dried methanolic extract (176.00 g) was subjected to fractionation using various solvents to obtain hexane (12.87 g), chloroform (37.66 g), dichloromethane (DCM) (42.32 g), and ethyl acetate (25.11 g) fractions. Commercial grade solvents were used for extractions and fractionations. The obtained fractions were dried and stored in the freezer for biological screening.

### 2.3. Animals

Healthy animals were purchased from Pakistan Council of Scientific and Industrial Research (PCSIR), Peshawar, and were maintained in the animal house of the Department of Pharmacy University of Swabi. The healthy mice of either sex were selected for in vivo pharmacological activities. Mice were fed with standard laboratory food and water ad libitum. All the experimental procedures were approved form the Ethical Committee (UOS/Pharm11), Department of Pharmacy, University of Swabi, Khyber Pakhtunkhwa, Pakistan.

### 2.4. Toxicological Study

An acute toxicological study was done on healthy mice according to the published methods [18]. The animals of each group were treated either with distilled water or extract/fractions at dose of 100, 250, 500, and 1000 mg/kg animal bodyweight. Each animal was observed for 6 h for gross behavioral changes after the administration of dose and then checked for mortality after 24 h.

### 2.5. Anti-Inflammatory Activity

The carrageenan-induced paw edema model was practiced for the evaluation of the anti-inflammatory effect of all the tested samples following a standard procedure [19]. Animals were classified into various groups (*n* = 6): negative control (animals were treated with distilled water, 10 ml/kg, IP), positive control (animals were treated with diclofenac, 5 mg/kg, IP), and test groups (animals were treated with 25, 50, 100, 200, 250, and 500 mg/kg, PO with various extract and fractions). After 30 minutes of the treatments, each animal's subplanter of the hind paw was injected with 1% carrageenan solution. The induced edema (inflammation) was quantified through plethysmometer just after administration of carrageenan and then after 1 h, 2 h, 3 h, 4 h, and 5 h. The percent attenuation in edema was quantified using the following formula.

(1) $$\begin{array}{c} \%\text{ inhibition}=\frac{A-B}{A}\times 100, \end{array}$$

where *A* and *B* represent the percent effect of negative control and tested group, respectively.

### 2.6. Analgesic Activity

The acetic acid-induced writhing test was used for evaluation of the antinociceptive effect of the extract and various fractions as per a previously reported method [20]. All the experimental healthy animals were fasted 2 h before the start of experiment. After 30 minutes of administration of distilled water (10 ml/kg), diclofenac (5 mg/kg), and extract/fractions (25, 50, 100, 200, 250, and 500 mg/kg), animals were treated with 1% acetic acid. After 5 min, the abdominal constrictions (writhes) were counted for 10 min. The attenuation in writhes during 10 minutes reflects the antinociceptive effect of the drugs.

### 2.7. Muscle Relaxation Activity

The crude extracts and various fractions were assessed for muscle relaxation potential by using the standard procedure [21].

#### 2.7.1. Inclined Plane Model

The inclined plane was used in screening and contained two plywood boards, which are connected in such a way that one board is aligned from the base while the other at 60^º^ from the base. All animals were distributed into several groups, and each group composed six animals (*n* = 6). Animals of every group were administered with distilled water (10 ml/kg), standard drug (diazepam, 1 mg/kg), and extract/fractions at 25, 50, 100, 200, 250, and 500 mg/kg. After the above administrations, animals were tested at 30 minutes, 60 minutes, and 90 minutes for the muscle coordination effect by allowing animals on the higher portion of the inclined plane for 30 seconds to hang or fall.

#### 2.7.2. Traction Model

The traction model was designed using a metallic wire covered with rubbers. The wire was linked with each other with the help of a stand, around 60 cm above the lab bench. The animals were divided in groups, treated, and tested at regular intervals. After that, all animals were suspended with wire to hang.

### 2.8. Sedative Activity

The sedative effect of extract and all fractions was performed as per standard methods [18]. The animals were divided into different groups (*n* = 6). The apparatus used in this screening comprised of an area of the white wood having 150 cm diameter bordered by stainless steel walls and was divided into nineteen squares by black lines. Then, the open-field apparatus was positioned inside of a light and sound attenuated room. Different groups of animals were fed with distilled water, diazepam, and extract/fractions as above. Thirty minutes after administration, all groups of animals were allowed to move from the center of the design box. The animals which crossed the maximum number of lines were recorded as “no sedation,” while animals with delayed movement were considered as “sedated.”

### 2.9. Statistical Analysis

The results have been displayed as mean ± standard error of the mean (SEM), and statistical significance was tested using one-way ANOVA. GraphPad Prism was used for statistical analysis.

## 3. Results

### 3.1. Toxicological Profile

The acute toxicology profile is given in Table 1. There was no mortality observed in any extract or fraction treated groups, even at the highest administered dose (1000 mg/kg bodyweight) during 24 hours of administration. However, at higher doses of various fractions, the locomotion was decreased, which is a determinant of sedative potential of the extract or fractions. The animals used for toxicity studies survived and did not exhibit any signs of delayed toxicity, even after 14 days observation.

### 3.2. Anti-Inflammatory Effect

**Figure 2:**
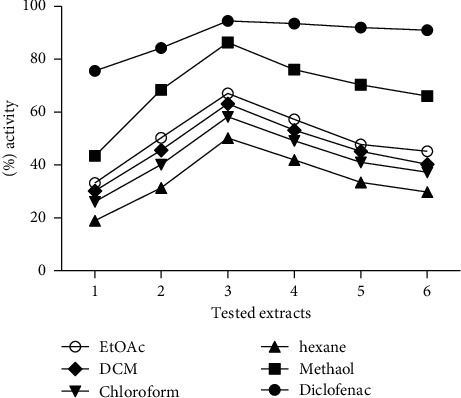
Anti-inflammatory screening of extract/fractions of *Syzygium cumini*.

The percentage anti-inflammatory effect is shown in Figure 2. The maximum percent inhibition effect was observed at the third hour of experiment in both standard and test samples. Among test samples, the methanolic crude extract demonstrated maximum inhibition (86.11%) at the third hour of experiment compared to other fractions. The percent anti-inflammatory potential of ethyl acetate (67.09%), dichloromethane (63.23%), and chloroform fractions (59.25%) of *S. cumini* was also significant, with least activity of *n*-hexane fraction.

### 3.3. Analgesic Effect

The analgesic potential of extract and fractions at various doses is given in Table 2. The crude extract of *S. cumini* dose-dependently inhibited the number of writhes at 50 and 100 mg/kg (*p* < 0.05). This effect was further potentiated at a dose of 250 and 500 mg/kg bodyweight (*p* < 0.001). The chloroform, DCM, and ethyl acetate fractions exhibited comparatively similar effect as methanolic extract. However, the *n*-hexane fraction was devoid of any analgesic effect. The analgesic activity of test samples was compared to the standard drug, diclofenac (5 mg/kg).

### 3.4. Muscle Relaxant Effect

The muscle relaxant effect of *S. cumini* extract and various fractions is given in Table 3. In both the inclined plane and traction models, a uniform effect was observed. The effect was measured at 30, 60, and 90 minutes later after sample administration. A time and dose-dependent effect was seen with extract and various fractions except for *n*-hexane. The maximum muscle relaxant effect of all tested plant samples was recorded at a dose of 500 mg/kg bodyweight, where the percent inhibition for dichloromethane was maximum (82.34%), followed by chloroform fractions (71.43%), methanolic extract (70.91%), and ethyl acetate fraction (69.22%). The activity of all test samples was compared to the muscle relaxant effect produced by the standard drug diazepam (1 mg/kg bodyweight), which was considered 100%.

### 3.5. Sedative Effects

The sedative effect of methanolic extract and fractions of *S. cumini* is given in Table 4. A significant (*p* < 0.001) sedation was noticed at higher doses (250 and 500 mg/kg) of the crude extract and various fractions of *S. cumini* except for *n*-hexane fraction. The tested samples were compared to the sedative effect produced by 0.5 mg/kg bodyweight of diazepam, which was the standard drug applied in this study.

## 4. Discussion

Natural products as therapeutic agents are the emerging, safe, and effective options in the modern era, especially in the developing world [22, 23]. Plant extracts as well as their isolated constituents are gaining popularity as therapeutic agents, e.g., ivy leaf (antitussive) and silymarin (hepatoprotective). Considering the promising efficacy and significant safety profile of natural products as therapeutic agents, the researchers got interested in screening these potential medicinal plants to validate their folkloric uses and claims [24–26]. In the current research work, the crude extract and various fractions of *S. cumini* were tested for various pharmacological activities. *S. cumini* is traditionally used for various inflammatory, painful conditions, sedation, and muscle relaxant [27–29]. The toxicity profile of *S. cumini* exhibited slight sedation and cessation of locomotion at a higher dosage of 500 mg/kg with zero mortality at maximum applied dose of 1000 mg/kg. The safe dose range for in vivo activities of the crude extracts and fractions was ascertained to be 25–500 mg/kg bodyweight. The tested *S. cumini* extracts and various fractions significantly attenuated edema induced by carrageenan. Crude methanolic extract of *S. cumini* inhibited paw edema more effectively followed by ethyl acetate, dichloromethane, chloroform, and n-hexane fractions, respectively. The analgesic activity of *S. cumini* extract exhibited dose-dependent analgesia with the same pattern of activity across various fractions, i.e., more effective methanolic extract followed by ethyl acetate, dichloromethane, chloroform, and n-hexane fractions, respectively. The inflammation induced by carrageenan is related to the release of various inflammatory mediators such as prostaglandins (PGs), bradykinin, and histamine [30]. Among these mediators, majorly, PGs are responsible for induction of pain, inflammation, and fever [31]. *S. cumini* probably inhibits cyclo-oxygenase (COX) and thus blocks the production of PGs and other intracellular cascade that leads to pain, inflammation, and pyrexia. In addition to the inhibition of PGs, *S. cumini* also exhibited sedative and muscle relaxant effects, which reflects its capability to modify some other pharmacodynamics pathways. *S. cumini* extracts exhibited potent muscle relaxant and sedative effects. The sedative and muscle relaxant effects suggested that the chemical constituents of *S. cumini* keep the anion neuronal channels open, especially the chloride channels, which leads to the depression of the central nervous system. The induction of anion influx is mostly related to stimulation of GABA (gamma aminobutyric acid) receptors, thereby hyperpolarizing the neuronal membrane via elevated chloride influx. These results further suggest that the crude extract or fractions might accelerate the action of GABA neurotransmitters, which is responsible for sedation and muscle relaxant effects [32]. However, further mechanistic studies should be conducted on various isolated constituents of *S. cumini* to know the exact mechanism of its activities.

## 5. Conclusion

*S. cumini* is a folklore medicinal plant traditionally, which is used in various health conditions. The present study substantiates the folklore medicinal claims about *S. cumini* as an anti-inflammatory, analgesic, sedative, and muscle relaxant and concludes that *S. cumini*, especially its crude methanol extract, has a significant role as anti-inflammatory, analgesic, sedative, and muscle relaxant. This study provides leads for researchers to isolate new and novel compounds from *S. cumini*, which have the biological potential for the treatment of various diseases.

## Figures and Tables

**Table 1:** Toxicological profile of crude extract and various fractions of *Syzygium cumini* in open-field screening.

Treatment	Doses (mg/kg)	Number of died animals/6	% mortality	Gross behavior changes
Normal saline	10 ml/kg	0/6	—	—

Methanolic extract	100	0/6	—	—
	250	0/6	—	—
	500	0/6	—	Inactive
	1000	0/6	—	Inactive

Hexane	100	0/6	—	—
	250	0/6	—	—
	500	0/6	—	—
	1000	0/6	—	—

Chloroform	100	0/6	—	—
	250	0/6	—	—
	500	0/6	—	Inactive
	1000	0/6	—	Inactive

DCM	100	0/6	—	—
	250	0/6	—	Inactive
	500	0/6	—	Inactive
	1000	0/6	—	Inactive

Ethyl acetate	100	0/6	—	—
	250	0/6	—	Inactive
	500	0/6	—	Inactive
	1000	0/6	—	Inactive

**Table 2:** Analgesic potential of crude extract and various fractions of *Syzygium cumini*.

Tested samples	Dose (mg/kg)	No. of writhes
Normal saline	10 mL/kg	61.098 ± 2.00

Diclofenac sodium	5	29.32 ± 2.20^∗∗∗^

Methanolic extract	25	44.09 ± 1.88
	50	39.34 ± 1.93^*∗*^
	100	34.43 ± 2.19^*∗*^
	250	29.98 ± 2.00^∗∗∗^
	500	25.09 ± 2.06^∗∗∗^

*n*-Hexane	25	62.65 ± 2.65
	50	57.21 ± 2.12
	100	51.32 ± 2.08
	250	45.54 ± 1.90
	500	40.32 ± 1.87

Chloroform	25	53.09 ± 2.12
	50	48.09 ± 3.01
	100	42.09 ± 2.98
	250	36.09 ± 2.06^*∗*^
	500	30.98 ± 2.43^∗∗∗^

DCM	25	41.34 ± 1.66
	50	35.66 ± 1.76^*∗*^
	100	29.11 ± 2.09^∗∗∗^
	250	23.09 ± 2.87^∗∗∗^
	500	18.34 ± 2.08^∗∗∗^

Ethyl acetate	25	46.09 ± 1.56
	50	39.34 ± 1.32^*∗*^
	100	33.98 ± 1.34^*∗*^
	250	27.98 ± 1.34^∗∗∗^
	500	22.87 ± 1.76^∗∗∗^

^*∗*^ *P* < 0.05; ^∗∗^*P* < 0.01, ^∗∗∗^*P* < 0.001.

**Table 3:** Muscle relaxant effect of crude and various fractions of *S. cumini*.

Group	Dose (mg/ml)	Inclined plan model (%)			Traction screening (%)		
		30 min	60 min	90 min	30 min	60 min	90 min
Distilled water	10 mL/kg	0.0	0.0	0.0	0.0	0.0	0.0

Diazepam	1	100	100	100	100	100	100

Methanolic extract	25	35.65	44.23	53.09	34.80	43.00	52.36
	50	41.54	50.98	59.23	42.43	51.11	60.32
	100	45.21	54.87	63.12	44.21	53.87	64.04
	250	48.23	57.54	66.32	47.23	56.98	67.06
	500	52.00	61.23	70.91	51.87	60.08	69.98

*n*-Hexane	25	12.22	20.03	29.98	11.30	19.76	30.70
	50	16.17	25.66	33.54	17.00	26.23	34.09
	100	21.28	28.23	37.11	20.32	27.06	36.60
	250	24.98	33.43	41.09	23.37	30.32	40.09
	500	28.87	36.98	44.21	27.43	35.21	43.08

Chloroform	25	30.11	39.43	48.94	29.01	38.06	47.66
	50	36.32	45.77	54.76	35.88	44.98	53.56
	100	42.12	51.43	60.70	41.54	50.32	59.54
	250	47.43	56.32	65.83	46.43	55.32	64.76
	500	53.98	62.87	71.43	52.98	61.90	70.03

DCM	25	38.08	47.51	56.01	37.50	46.70	55.91
	50	44.65	53.57	62.43	45.00	54.01	63.07
	100	50.23	59.60	68.22	51.89	60.90	67.32
	250	57.42	66.68	75.19	56.86	65.02	74.00
	500	64.21	73.76	82.34	63.01	72.98	81.03

Ethyl acetate	25	27.43	36.21	45.45	26.76	35.08	44.14
	50	33.60	42.98	51.12	32.86	41.16	50.87
	100	39.09	48.76	57.98	40.00	49.83	58.72
	250	45.43	54.23	63.09	44.87	53.98	62.43
	500	51.66	60.65	69.22	50.94	59.98	68.88

**Table 4:** Sedative effect of crude and various fractions of *Syzygium cumini* in open-field screening.

Samples	Dose (mg/ml)	Number of lines crossed
Control	5 ml	130.00 ± 3.65

Diazepam	0.5	9.23 ± 0.55^∗∗∗^

Methanolic extract	25	88.32 ± 3.01
	50	82.04 ± 2.79
	100	75.54 ± 2.70^*∗*^
	250	67.09 ± 2.65^∗∗^
	500	60.21 ± 1.40^∗∗∗^

*n*-Hexane	25	112.09 ± 2.09
	50	108.32 ± 2.30
	100	101.87 ± 2.44
	250	95.32 ± 2.54
	500	89.01 ± 2.05

Chloroform	25	80.23 ± 2.13
	50	74.98 ± 2.12
	100	68.23 ± 2.30
	250	62.09 ± 2.34^∗∗∗^
	500	57.12 ± 2.88^∗∗∗^

DCM	25	43.98 ± 2.11^*∗*^
	50	37.01 ± 2.73^∗∗^
	100	32.32 ± 2.60^∗∗∗^
	250	26.21 ± 2.43^∗∗∗^
	500	20.90 ± 2.00^∗∗∗^

Ethyl acetate	25	75.8 ± 3.00
	50	69.65 ± 2.83
	100	63.87 ± 2.50^*∗*^
	250	57.23 ± 2.12^∗∗^
	500	52.56 ± 1.82^∗∗∗^

^*∗*^ *P* < 0.05; ^∗∗^*P* < 0.01. ^∗∗∗^*P* < 0.001.

## Data Availability

The data used to support this study are included within the article.
